# Polarized Cells, Polarized Views: Asymmetric Cell Division in Hematopoietic Cells

**DOI:** 10.3389/fimmu.2014.00026

**Published:** 2014-02-03

**Authors:** Kim Pham, Faruk Sacirbegovic, Sarah M. Russell

**Affiliations:** ^1^Immune Signalling Laboratory, Peter MacCallum Cancer Centre, East Melbourne, VIC, Australia; ^2^Centre for Micro-Photonics, Faculty of Engineering and Industrial Sciences, Swinburne University of Technology, Hawthorn, VIC, Australia; ^3^Department of Pathology, University of Melbourne, Melbourne, VIC, Australia; ^4^Sir Peter MacCallum Department of Oncology, University of Melbourne, Melbourne, VIC, Australia

**Keywords:** cell polarity, asymmetric cell division, immunological synapse, scribble complex, cell fate

## Abstract

It has long been recognized that alterations in cell shape and polarity play important roles in coordinating lymphocyte functions. In the last decade, a new aspect of lymphocyte polarity has attracted much attention, termed asymmetric cell division (ACD). ACD has previously been shown to dictate or influence many aspects of development in model organisms such as the worm and the fly, and to be disrupted in disease. Recent observations that ACD also occurs in lymphocytes led to exciting speculations that ACD might influence lymphocyte differentiation and function, and leukemia. Dissecting the role that ACD might play in these activities has not been straightforward, and the evidence to date for a functional role in lymphocyte fate determination has been controversial. In this review, we discuss the evidence to date for ACD in lymphocytes, and how it might influence lymphocyte fate. We also discuss current gaps in our knowledge, and suggest approaches to definitively test the physiological role of ACD in lymphocytes.

## Introduction

An effective immune response relies on the coordination of signals to control major cell fate checkpoints such as proliferation, differentiation, survival, and death. While many key players, including surface molecules, transcription factors, and cytokines have been identified to be important for immune cell fate control, it is still not clear how these signals are integrated during the differentiation and function of B and T cells. These questions of how signals are orchestrated during cell fate determination have been particularly well addressed in progenitor cells of the developing worm and fly. In these two organisms, cell fate is strongly influenced by the asymmetric distribution of fate determinants into the two daughters of a dividing cell, known as asymmetric cell division (ACD) (1). ACD involves the differential partitioning of protein, mRNA, microRNA, and other cellular constituents into the two daughter cells. Therefore, ACD imparts differential fates such as self-renewal, quiescence, proliferation, differentiation, and apoptosis. The mechanisms and consequences of ACD were initially studied in *Drosophila melanogaster* neuronal precursors, and *C. elegans* zygote formation, but have now been elucidated in many tissues, including those of mammals. In this review, we describe our current understanding of the mechanisms and consequences of ACD in cells of solid tissues, discuss the evidence that similar processes might apply in hematopoietic progenitor cells, B cells, and T cells. We also discuss, what will be required to determine whether there are physiological roles for ACD in lymphocyte development, function, and disease.

## The Role of ACD in Solid Tissues

**Figure 1:**
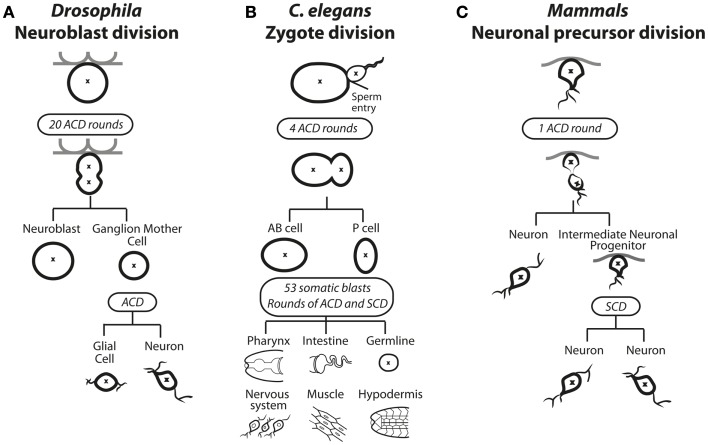
**Asymmetric cell division in solid tissues of (A) *Drosophila*, (B) *C. elegans*, and (C) Mammals**. **(A)** In *Drosophila*, selected neuroblasts undergo up to 20 rounds of asymmetric cell division (ACD). The asymmetric distribution of polarity and cell fate determinants causes spindle asymmetry to result in a large self-renewing neuroblast cell and a smaller ganglion mother cell (GMC). The GMC undergoes a subsequent ACD to produce a glial cell and a neuron. **(B)** ACD during zygotic division in *C. elegans*. The site of sperm entry serves determines the asymmetric distribution of polarity and cell fate determining proteins as well as spindle asymmetry. During the embryonic stage four rounds of ACD results in the emerging anterior body (AB) and posterior (P) cells. During the larval stage, 53 somatic blasts undergo bursts of ACD and symmetric cell division (SCD), specifying all future posterior or soma fates in various tissues. **(C)** Neuronal precursor asymmetric division in mammals. The first asymmetric cell division produces a neuron and an intermediate neuronal precursor (INP), which undergoes a symmetric division to produce two neurons.

Homeostasis of stem cells frequently involves ACD, where a parent cell divides to generate a daughter cell identical to itself (“self-renewal”), as well as another daughter that is programed to proliferate, differentiate, or both (1). In some instances, the different fates of the two daughters can occur through stochastic responses in which each daughter has some probability of either self-renewing or adopting a different fate to maintain an appropriate balance of self-renewing and differentiating progeny on a population level. In other instances, the balance between self-renewal and differentiation is controlled at the single cell level by ACD. An example in which ACD controls the expansion and differentiation of the cells occurs in the developing *Drosophila* central nervous system (2) (Figure 1A). During development of the larval central nervous system, neuroblasts delaminate from the neurepithelium to undergo up to 20 rounds of ACD, each round creating another neuroblast (“self-renewal”) and a ganglion mother cell (GMC) that can further proliferate and differentiate to form mature neurons. Neuroblasts become quiescent during pupation but then re-enter the cell cycle and reinitiate ACD for further rounds of proliferation and differentiation (1). The limited set of neuroblasts therefore undergoes controlled ACD that contributes to the thousands of adult neurons and neuronal associated cells of the central nervous system.

ACD plays a dominant role in dictating fate in *C. elegans*, starting at the first zygotic division after fertilization, when the fertilized egg divides asymmetrically to produce an anterior AB cell and a posterior P1 cell (Figure 1B). Four rounds of ACD follow; each producing one daughter that contributes only to soma and the other only to the germline. Thus, ACD controls differentiation and influences the expansion of cells from one generation to the next (3). ACD also occurs in mammals during brain and gut development. During brain development, a burst of symmetric cell divisions (SCDs) increases the progenitor pool, then sequential ACD in the neurepithelium balance self-renewal with differentiation of cells committed to the neuronal lineage (Figure 1C) (4). During mammalian gut development, in particular the colonic crypt, there is a high turnover of tissue where up to 10^10^ mature gut cells are replenished using a balance of symmetric and asymmetric divisions (5, 6). Within the folds of epithelium lining the colon, crypt cells continually undergo ACD to self-renew and generate proliferative daughter cells that terminally differentiate and transiently populate the migrating compartment, then die. ACD in mammals has also been observed during the development and differentiation of muscle, mammary glands, and skin (7–12). The mechanisms guiding these decisions in mammals are not well understood, but many molecular players that were identified in *C. elegans* and *Drosophila*, as discussed in the next section, have also been implicated in mammalian ACD.

An interesting aspect of ACD is the varied extent of influence that has been observed in different developmental systems. ACD is absolutely required during zygotic development in *C. elegans*, where the molecular differences between the daughter cells directly specify their different fates (13–18). In contrast, ACD of *Drosophila* nervous system is not (or less) deterministic, as subsequent fate decisions are subject to influences from the microenvironment [reviewed in Ref. (19)]. In some instances, the primary molecular consequence of ACD is a difference in signaling between the two daughter cells. Rather than specifying the differentiation path for the two daughter cells, this merely ensures that the two daughter cells adopt different fates from each other in response to external influences (20, 21). Context can play another important role by controlling whether a cell divides symmetrically or asymmetrically. In contrast to the prescriptive pattern in *C. elegans*, where the early divisions are uniformly asymmetric, cell divisions in the mammalian developing nervous system can switch from symmetric to asymmetric to selectively expand specific cellular pools, or to generate more differentiated cell types as the need arises (22, 23).

## Molecular Regulation of ACD

**Figure 2:**
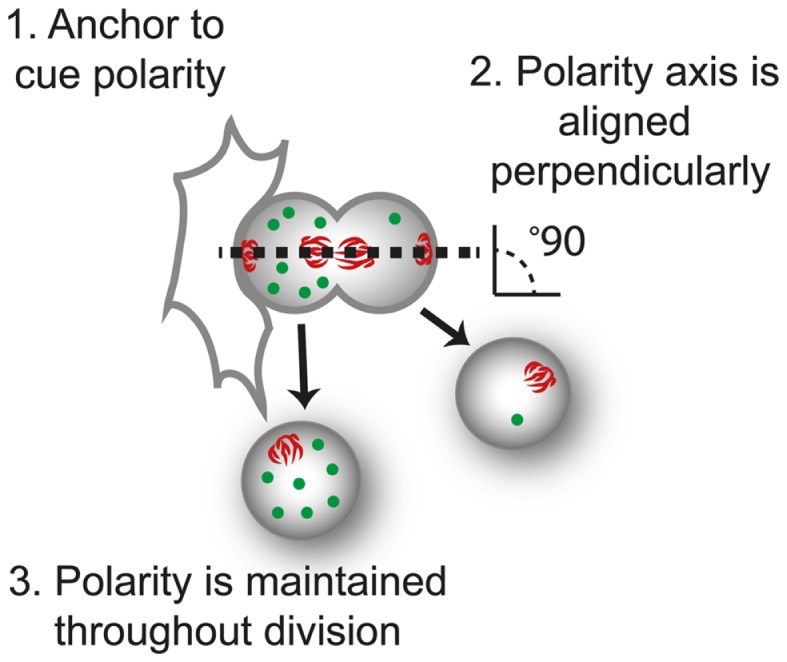
**The three requirements of asymmetric cell division**. For control of progeny proliferation, death, and differentiation during asymmetric cell division ACD, three requirements must be fulfilled; (1) an anchor to dictate the axis of polarity, in this case another cell; (2) the dividing cell is aligned along the axis of division, usually perpendicular to the anchor (perpendicular orientation shown by the alignment of mitotic spindle, red); and (3) that polarity of the protein (green) is maintained throughout division.

**Figure 3:**
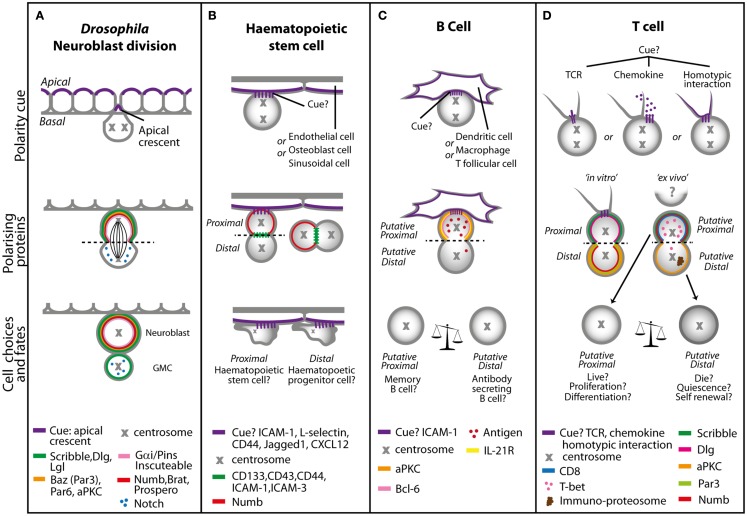
**Models of asymmetric cell division in (A) *Drosophila* neuroblasts, (B) hematopoietic stem cells, (C) B cells, and (D) T cells**. **(A)** In *Drosophila*, neuronal precursors delaminate from the neurepithelium to undergo ACD. The polarity cue is the apical crescent, and during early division duplicated centrosomes rotate 90° to create the distinct apical and basal sides that are mediated by the Scribble and Bazooka polarity protein complexes. During late division, the coordination of the spindle length by Gα_i_ signaling and proteins such as Inscuteable and Pins result in asymmetric distribution of cell fate determinants, such as Numb, Notch, Brat, and Prospero. The coordination and maintenance of signaling results in a self-renewing neuroblast cell and a ganglion mother cell (GMC). In cells of the hematopoietic system, multiple polarity cues can dictate asymmetric cell division. **(B)** Hematopoietic stem cells migrating in a stem cell niche in the bone marrow can receive adhesion, Notch or chemokine cues from surrounding endothelial, osteoblast, or sinusoidal cells, resulting in asymmetric distribution of cell fate determinants such as Notch and Numb (during attachment with the interacting cell or separately) to produce a self-renewing hematopoietic stem cell and a hematopoietic progenitor cell, which will go on to differentiate. In **(C)** B cells and **(D)** T cells the polarity cue might be through interaction with macrophages, other T cells and antigen presenting cells such as dendritic cells via adhesion, chemokine, or TCR molecules. This interaction sets an axis of division and asymmetric distribution of several surface molecules, antigen polarity, and cell fate determinants. In B cells daughters proximal to the interacting cells favor memory B cell fate, as well as more potent T cell activators and proliferators. Distal B cell daughters favor antibody secreting cell fate, with moderate T cell activating and proliferative capabilities. In the absence of ICAM-1, B cell fate is altered toward memory B cells at the expense of antibody secreting B cells. T cell daughters will inherit factors that will increase or decrease their propensity to adopt a variety of fates including that of a memory or effector T cell.

ACD involves three processes: (i) cellular cues to dictate the axis of polarity; (ii) opposing actions of polarity proteins to dictate molecular differences along this axis; and (iii) the alignment of the mitotic spindle with the polarity axis to maintain asymmetry during division (Figure 2). Many of the proteins involved in establishing polarity and aligning the mitotic spindle are evolutionarily conserved, but differences occur in the cues that dictate the orientation of polarity, the composition of the polarity modules, and the fate determinants that dictate the differences in the functional outcome in different cell types. Here, we focus on the *Drosophila* central nervous system to illustrate the principles of mutual antagonism and connectivity with the spindle pole that are required for ACD (Figure 3A).

Asymmetric cell division and segregation of cell fate determinants in *Drosophila* neuroblasts is regulated by the interactions between the Scribble and Bazooka (Par3 in mammals) polarity complexes. Through the interaction with the Gα_i_ complex, the Scribble and Bazooka complexes also coordinate the orientation of the mitotic spindle. During ACD, the Bazooka and Gα_i_ complexes are linked via an adaptor protein, inscuteable, and polarize to the apical cortex of the dividing cell (24–26). In addition, Dlg (from the Scribble complex) binding to the plus-end directed microtubule motor protein Khc-73 (Kinesin heavy chain 73) and Pins regulates the positioning of the Gα_i_ complex (27, 28).

The mechanism by which the Par3/Bazooka and Scribble complex delineate the two poles of the cell is not yet clear, but it is thought to involve the regulation of aPKC phosphorylation activity by Lgl. The activity of aPKC is inhibited when it is in a complex with Par6 and Lgl (part of the Scribble complex) (17, 18). During mitosis, Par6 is phosphorylated, which relieves the repression of aPKC activity and allows aPKC to phosphorylate and release Lgl from the complex. This in turn allows the restriction of aPKC localization to one side of the cell cortex, where it is free to phosphorylate and release the cell fate determinants such as Numb from that side of the cortex (29, 30). One key observation is that while proteins of the Par3/Bazooka and Scribble complex localize at the apical side during early neuroblast division, some members of these complexes disperse cortically at telophase (6). Moreover, mutations in *scribble* or *lgl* do not affect Dlg polarization, but Dlg is required for the cortical recruitment and polarization of both Scribble and Lgl (6).

The role of ACD in steering fate determinants preferentially into one daughter cell is illustrated by the phenotypes in *Drosophila* in which the polarity and spindle regulators are mutated. For instance, the loss of *lgl* results in loss of asymmetric recruitment of fate determinants such as Numb in neuroblasts (6, 31, 32). Mutations in *scribble, dlg*, or *lgl* lead to mislocalization of multiple basal cell fate determinants and disrupt orientation of the mitotic spindles, which results in perturbed cell size and decreased GMC fate specification. In contrast to *lgl* mutations, mutations in *aPKC* lead to reduced neuroblast proliferation (33). Interestingly, the neuroblast hyperproliferation and mislocalization of cell fate determinants that is associated with *lgl* mutant flies can be partially rescued when crossed with *apkc* mutant flies (34), indicating that interactions between aPKC and Lgl specify respective cell fates. Other studies have also identified that Numb is a substrate for aPKC, and that aPKC-mediated phosphorylation is critical for the asymmetric segregation of Numb and the specification of neuroblast fate (29, 30, 35). Other genetic and biochemical assays show that Numb directly binds to the intracellular domain of the cell fate determinant, Notch. It is postulated that Numb acts as a negative regulator by mediating Notch degradation via endocytic pathways mediated by alpha-Adaptin, a component of the clathrin associated endocytosis pathway, which targets proteins for endocytosis (36, 37).

## Does ACD Occur in the Hematopoietic System?

As with the numerous fate choices governing *Drosophila* neuroblast fate, all cells of the hematopoietic system make fate decisions related to differentiation, proliferation, death, and self-renewal. It would seem reasonable that cells of the hematopoietic system should also adopt the process of ACD as a means of controlling such decisions. Elucidating a possible role for ACD in fate determination of hematopoietic cells, however, has been slower than elucidating the role for ACD in cells of solid tissues. First, the seminal work in model organisms such as *C. elegans* and *Drosophila* is more readily applicable to solid tissues in mammals, where the cue and fate determinants are often conserved across species (Table 1). For instance, elucidation of ACD in mammalian neurons has benefited directly from findings in *Drosophila*, but there is no *Drosophila* parallel to guide studies in the hematopoietic system. Second, most progress has been made in systems, where the entire developmental program can be tracked and correlated with molecular behavior during and subsequent to each cell division. This has been the case with *C. elegans*, where more than three decades of research effort has been invested into exploring the mechanisms by which ACD regulates development of the *C. elegans* embryo by time lapse microscopy (38). Similarly in *Drosophila*, two decades ago direct observations that Numb was recruited into the GMC upon division were reported, and that levels of Numb dictate neuronal differentiation (39).

**Table 1:** **Known functions of polarity proteins in. lymphocytes**.

Polarity protein	Known phenotypes		Lymphocyte phenotype	
PAR-1	•	Mutation (*C. elegans*): controls spindle positioning (12)	•	Dominant negative mutation (T cells): loss of Par1b polarization and TCR-induced MTOC polarization (111)
PAR-1	•	Mutations (*Drosophila*): failed oocyte polarity. Phenotypes can be rescued by expressing ParN1 isoforms (121)	•	Loss (B and T cells): normal B and T cell development. CD4^+^ T cells exhibit higher TCR activation, B cell T-dependent and T-independent responses are altered, suggesting autoimmunity (112)
Par1b/MARK2/EMK

PAR-3	•	Mutation (*C. elegans*): posterior shift during P0 asymmetric cell division (15)	•	N/A. Par3 is excluded from the T cell uropod, and may localize transiently to the synapse during immunological synapse formation (78)
Bazooka			
Par3, ASIP, PARD3	•	Mutation (*Drosophila*): loss of apical cue for Inscuteable localization in asymmetrically dividing neuroblasts (25)
	•	Loss (*Drosophila*): disruption of embryo basolateral membrane polarity during mid-gastrulation (122), mislocalization of Numb, and planar symmetry of pI cell during sensory organ precursor ACD (123) failed oocyte ACD (124)
	•	Removal/ectopic expression (mammalian neocortex): reduction of ACD and transformation of glial progenitor fate (57)	

PAR-6	•	Mutation (*Drosophila*): failure of oocyte differentiation (45)	•	Overexpression of Par6 N-terminal aPKC interacting domain reduces T cell uropod formation (106)
Par6	
Par6 α, β, and γ/PARD6 A, B, and G	

Pkc-3	•	Mutation (*C. elegans*): posterior shift during P0 division (15)	•	Overexpression/dominant negative mutation (T cells): randomizes F-actin distribution, impairs uropod formation, motility, defects in T cell scanning (106, 127)
Atypical PKC	•	Knockdown (*Drosophila*): reduced cell proliferation in both neuroblasts and epithelia (34, 125)
PKCι/λ and ζ	•	Loss (*Drosophila*): loss of AB cell polarity (126), failed oocyte ACD (124)	•	Drug inhibition of aPKC/Par6 interaction (T cells): defective Numb localization during T cell ACD (94)
			•	Loss: delay in secondary lymphoid organ formation (66), no naive T cell defects, Th2 differentiation but not Th1 differentiation is impaired, inhibition of ovalbumin induced allergic airway disease (108)
			•	Combined PKCι/λ/ζloss (HSC’s, B and T cells): normal HSC self-renewal, engraftment, differentiation, interaction with the bone marrow microenvironment, polarization, self-renewal. Normal mature B cells and T cells numbers (56)

Crb-1/Crb-like	•	Mutations (*Drosophila*): loss of epithelial cell morphology in the ectoderm (128)	•	N/A
Crumbs
Crumbs1-3	•	Ectopic expression of Crumbs3 (mammalian epithelia): loss of tight junction formation and intracellular polarity (129)	

C01B7.4/tag-117	•	Loss (single and combined with Crumbs loss, *Drosophila*): disruption of the establishment and maintenance of epithelial morphology in the embryo (130), disrupted embryonic basolateral membrane polarity during mid-gastrulation (122)	•	Knock down (T cells): suboptimal T cell activation and proliferation. Strongly localized to the Golgi apparatus, and is mislocalized upon Bref A (Golgi disrupting) treatment (55)
Stardust
Pals1/MPP5
	•	Knockdown (mammals): loss of MDCK cell polarization in confluent cellular monolayers (129)	

Let-413	•	Mutations (*Drosophila*): reduced neuroblast size, increased ganglion mother cell size, defects in targeting cell fate determinants, and altered spindle asymmetry (6)	•	Knockdown (T cell line): prevention of TCR receptor polarization in response to antigen presentation, reduction in migration due morphological changes resulting in reduction of uropod formation (78)
Scribble
Scribble
	•	Loss (*Drosophila*): defects in junction formation and epithelial organization. Hyperproliferation, formation of solid tumors in imaginal disk and follicular epithelia (121)	•	Knockdown (thymocyte): defects in cell–cell clustering and maturation (77)
			•	Loss: (T cells): altered pERK signaling in T cells but responses to influenza infection are intact. (B cells): delayed B cell proliferation, but T dependant and T independent activation are normal (63)

Dlg-1	•	Mutation (*Drosophila*): imaginal disk hyperproliferation, tumorogenesis, and transform into solid tumors (131), defects in neuroblast size and mitotic spindle asymmetry (6)	•	Overexpression (T cells): attenuates basal and Vav1-induced NFAT reporter activation (92)
Disks large
Dlg 1–4			•	Knockdown (T cells): enhances both CD3- and superantigen-mediated NFAT activation (89, 90, 92). Accumulation of actin at the T cell synapse, altered production of Th1 and Th2 cytokines (68)
			•	Loss (thymocytes and T cells): normal T cell development (63, 68). Variable defects in mature CD4^+^ T cell differentiation (68, 92) Normal TCR-induced early phospho-signaling, actin-mediating events, proliferation, (68)

Lgl-1	•	Mutation (*Drosophila*): defects in neuroblast apical cell and spindle pole size resulting in symmetric or inverted neuroblast cell divisions. Loss of polarity in tissues that leads to overproliferation and tumor growth (5, 132)	•	Loss (HSC): increase in HSC numbers, cycling, increased HSC repopulation capacity and competitive advantage after transplantation (58)
Lethal Giant Larvae
Lgl1, Lgl2	•	Loss (mammals): neural progenitor cells fail differentiation, fail to exit cell cycle, then over proliferate and result in neural ectodermal tumors. Mislocalization of Numb in the neuroectoderm of the tumors (133)	

*Homologs:* 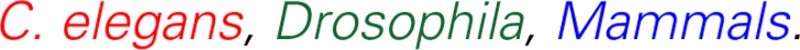

Frustratingly, longitudinal analyses *in vivo* are still not conceivable for ACD in the hematopoietic system because morphology is less informative (size differences do not indicate subsequent fates) and the cells are highly motile (cannot be tracked *in vivo* over generations). Also, differentiation generally occurs gradually over a longer time period. Instead, we have relied so far on correlative findings, each of which contributes to, but does not definitively prove the notion that ACD in cells of the hematopoietic system, including lymphocytes impacts upon cell fate decisions. Experiments to explore a role for ACD in cells of the hematopoietic system such as HSC, and lymphocytes such as B cells and T cells, so far have involved seeking three lines of evidence:

1. Evidence of a bifurcation in cell fate in the daughter cells of a dividing hematopoietic cell;
2. Evidence of asymmetry in dividing hematopoietic cells (defining the polarity cue and the fate determinants that are asymmetrically distributed);
3. Evidence of fate alterations upon disruption of the control of ACD.

The burden of proof lies in trying to combine these approaches to demonstrate in the same system that both ACD and fate bifurcation occurs, that ACD is associated with cell fate decisions by the daughter cells, and that both are disrupted by deregulation of a cell polarity regulator.

## ACD in HSC

As with all stem cells, it is well accepted that blood homeostasis involves a bifurcation in HSC fate whereby one daughter of an HSC is a copy of the parent (self-renewal) and the other expands and differentiates to give rise to the many blood lineages. There is growing acceptance that HSC may also undergo ACD to regulate fate choices, and the cues that might regulate ACD have been well established (38, 40). HSCs interact with a niche within the bone marrow, fetal liver, and peripheral blood, which could provide polarity cues to mediate ACD for fate determination (41). For example, the osteoblasts in bone marrow can express Notch ligands such as Jagged-1, adhesion molecules such as ICAM-1/LFA, L-selectins, and CD44, and also express chemokines such as CXCL12 (3) (Figure 3B). Initial evidence that ACD might occur in HSC came from Reya and colleagues, who provided preliminary evidence of asymmetric distribution of Numb in HSC treated with nocodozole to block cells in mitosis. This observation is difficult to reconcile with findings that hematopoiesis seems completely normal in Numb and double *numb*–*numblike* conditional mutants (42, 43) and in mice with deletion of Numb-like combined with hypomorphic alleles of *numb* that produce 5–10% of Numb protein (44–46). Possible explanations for this apparent discrepancy include: incomplete deletion of Numb [recombination at the Numb locus can be context specific (47), and an incomplete deletion of Numb/Numb-like might still leave a few wildtype hematopoietic progenitors to undergo normal lymphoid lineage development, as one or few HSC can repopulate the entire hematopoietic system (48, 49)]; or that the Numb allele under investigation deletes only exons 5 and 6 and so might not act as a complete null in hematopoietic tissues (50). Another explanation might relate to the notion that ACD, rather than impacting the levels of proteins in individual cells, might create differences in expression levels between neighboring cells to influence fate (51). In this case, mutant alleles that could not segregate asymmetrically might be more informative than mere deletion of the gene. Regardless, an exciting finding from the Reya study was that by using a fluorescent reporter of Notch signaling and time lapse imaging of paired daughters, they showed that HSC can produce daughters with different Notch signaling capacities, and that the proportion of HSC with differential Notch signaling in the daughters differed depending upon the stromal cells with which they were cultured (52).

Work from the Sauvageau laboratory using gain-of-function *in vitro* and *in vivo* assays found a component of the endosomal AP-2 complex, alpha-Adaptin (encoded by the Ap2a2 gene), to endow *in vivo* proliferative advantage and an increase in *in vitro* HSC maintenance (53). Given that alpha-Adaptin is also important for ACD in *Drosophila* neuroblasts and sensory organ precursors, these findings suggest that mechanisms of fate determination through ACD could be evolutionarily conserved in HSC. In support of this notion, time lapse imaging of HSC containing fluorescently-tagged alpha-Adaptin showed asymmetric inheritance in approximately 50% of HSC divisions. Knockdown of alpha-Adaptin did not affect HSC proliferation, differentiation, homing or apoptosis, despite alpha-Adaptin mRNA expression being fourfold to eightfold higher in long-term HSC than in intermediate term HSC. Interestingly, alpha-Adaptin and Numb were not co-localized in HSC, unlike in *Drosophila* neuronal precursors (54), highlighting possible divergent mechanisms of cell fate control.

Besides these two studies, remarkably little is known of the mechanisms by which ACD of HSC might be regulated. Perhaps because cell division of an HSC is, by definition, an extremely rare event, there has been little imaging to determine what molecules are localized asymmetrically at the time of division. The role of polarity proteins in controlling cell fate in HSCs has not yet given a strong indication of the importance of ACD in hematopoiesis. While RNA interference of the Par3 complex proteins, Par6 and PKCζ can impair HSC repopulation (55), single and double knockouts of PKCζ and PKCι/λ have no effect on HSC function, in primary and secondary engraftment (56). Adding to this confusion is that aPKC phosphorylates and regulates the Scribble complex protein, Lgl, and loss of Lgl1 leads to enhanced engraftment and better HSC repopulation capacity due to increased proliferation (57). The evidence to date is therefore suggestive rather than definitive that ACD might control aspects of HSC self-renewal and differentiation.

## ACD in B Cells

### Are there bifurcations in B cell fate that could be influenced by ACD?

B lymphocyte development involves fate choices such as proliferation, self-renewal, and differentiation to result in the formation of memory B cells, and plasma cells that produce antibodies of unique specificity (58). Duffy and colleagues recently produced the most exhaustive study to date to determine whether the daughter cells of a dividing B cell exhibit asymmetric fates (59). Time lapse analysis of differentiation, death, and time to next division imaged from one cell division to the next, showed that daughters from B cell divisions stimulated by interleukin-4 (IL-4) and IL-5 largely undergo symmetrical fates. Interestingly, a small proportion of B cell divisions displayed asymmetric cell fates in which one daughter died and the other survived. The authors determined that the discrepancy in fate observed in this fraction was not a result of asymmetric programing but of the internal competition for fates within each cell. In this model, which is well supported by examples across many species, each cell is programed for “time to die” and “time to divide,” and these times are reset upon each cell division (60–62). Similarly, in the B cell study, the “time to die” and “time to divide” were set very close together, such that in some instances the two daughter cells from a single B cell had an equal probability of adopting either fate. This study argues against ACD controlling B cell fate. It should be noted that the symmetrical fate observed here was in the context of soluble activating factors, rather than a directional cue, so does not discount a role for ACD in other forms of B cell activation. In line with the Duffy et al. study, our time lapse analysis of B cells stimulated with another soluble agent lipopolysaccharide also argued against ACD (63).

### Is there evidence of polarity in dividing B cells?

In support of the notion that B cells could receive instructional cues through engagement with dendritic, macrophage, or T helper cells to dictate downstream fates via ACD, Barnett and colleagues explored polarity in the germinal center (64). Dividing B cells within the germinal center asymmetrically localized the transcription factor Bcl6, the receptor for IL-21, and the polarity protein PKCζ (64). Asymmetry of these proteins during division required constant signaling through contact with antigen presenting cells, possibly via adhesion through LFA-1/ICAM-1. In ICAM-deficient mice, B cells did not efficiently polarize Bcl6 or PKCζ, and showed a defect in the number of antibody secreting plasma cells. The evidence of polarity at division, and the correlation of loss of polarity with cell fate differences caused by loss of ICAM-1 (which might have many non-polarity related effects), is compatible with the notion that germinal center B cells undergo ACD to influence cell fate, but further quantification and evidence that the polarity is controlled rather than stochastic, is required to confirm this.

In a separate study, multi-photon microscopy of explanted lymph nodes showed that B cells acquired antigen from macrophages in a polarized manner *in vivo*, and that the acquired antigen could accumulate preferentially in one daughter cell after B cell division (65). Antigen asymmetry persisted for up to three rounds after B cell division, and, statistical modeling predicted that up to 25% of B cells undergo asymmetric inheritance of antigen. There was no evidence for involvement of a polarity cue, or of the molecules involved in polarity, suggesting that the asymmetry of antigen inheritance was more likely a stochastic response than a result of ACD (Figure 3C).

### What is the phenotype of polarity-deficient B cells?

As with the fate tracking information above, analysis of polarity-deficient B cells provides evidence both for and against a role for ACD (Table 1). Mice deficient in PKCζ exhibited subtle delays in B cell development, but these defects were normalized in older mice (66). The B cells from 4- to 6-week-old PKCζ-deficient mice also show severe defects in *in vitro* proliferation, enhanced ERK signaling in response to B cell receptor cross-linking (but not in response to non-B cell receptor stimuli), could mount a normal T independent humoral response *in vivo*, and showed slight defects in T-dependent humoral responses (67). B cell development is grossly normal in the absence of Dlg1 (63, 68), Lgl1, and Scribble (63), although a recent paper suggests that Dlg1-deficient B cells, like PKCζ-deficient B cells, exhibit developmental defects in young mice that are rescued in older mice (69). Knockdown of Dlg1 (also called SAP97) in B cells *in vitro* impaired the formation of the immunological synapse and inhibited BCR-dependent responses (70). Scribble-deficient mice have intact *in vitro* and *in vivo* humoral responses to activation and infection respectively, but again show perturbed kinetics of ERK phosphorylation (63) as previously seen in epithelial tissues when Scribble is depleted (71–73). Combined, these data do not provide compelling support for a role for ACD in B cell development or responses. The hints of B cell phenotypes in some knockouts, and the observations that these phenotypes diminish with age, suggest that compensatory mechanisms that might make combined or more acute deletions necessary to determine the role of ACD in B cell development and function.

## ACD in T Cells

### ACD in thymocytes

A small number of observations suggest that polarity proteins, and perhaps ACD, might also play a role in developing T cells. Nearly 50 years ago, the proportions and kinetics of proliferation of three types of thymocytes, as distinguished by their size, were assessed using autoradiographic analysis of tritiated thymidine uptake, and the data fit a requirement for ACD (74). This was followed by microscopic evidence of asymmetry at division, as defined by differences in the cytoplasmic or nuclear size in the two daughters in several species including the mouse (75). The involvement of polarity proteins was shown in *in vitro* interactions between thymocytes and dendritic cells, where Dlg1 was rapidly polarized to the synapse following TCR activation (76). Pike and colleagues demonstrated that *in vitro* DN3 thymocyte development was perturbed by knockdown of Scribble, with an accumulation of DN3 thymocytes and inefficient double positive CD4^+^CD8^+^ thymocyte generation (77). Interestingly, depletion of Scribble affects DN3 thymocyte clustering by limiting the polarization of the integrin ICAM-1/LFA-1 (77, 78). In a study by Aguado and colleagues, the transgenic expression of wildtype or dominant negative forms of Numb result in altered DN3 thymocyte pre-TCR signaling, proliferation, and differentiation (79). Asymmetry of Numb was also proposed by this group as a mechanism for these signaling and fate differences, but asymmetry was not rigorously assessed. Taken together, these studies provide hints that polarity and cell fate proteins are important for aspects of T cell development and downstream fate choices. Careful analysis of protein localization at division, and correlation of any asymmetry with alterations in fate, will be required to elucidate a possible role for ACD in thymocyte differentiation.

### Are there bifurcations in T cell fate that could be influenced by ACD?

Perhaps the most studied and most controversial aspect of lymphocyte ACD is in mature T cells. In part, the controversies are due to the elusive nature of the fate choices that a naïve T cell makes upon stimulation by an antigen presenting cell. CD4^+^ cells can differentiate along many pathways upon stimulation (80), but will not be discussed in detail here as the role of ACD in CD4^+^ differentiation has not been extensively pursued. CD8^+^ naïve T cells give rise to both effector and memory progeny, and many subpopulations within these categories. A bifurcation of fate decisions by the two daughters of a naïve CD8^+^ cell would be an appealing explanation for how one naïve T cell can yield both effector and memory populations (81). Despite the wealth of literature on the subject, it is still not clear exactly when the two lineages arise from a naïve T cell, and for instance whether (and how far) memory cells progress down the effector differentiation pathway before committing to a memory fate (82–84).

Several recent papers provide support for the notion that fate is controlled at many stages during T cell activation, including the time of first division, when ACD could play a role. Three recent studies assess the progeny of individual CD8^+^ clones *in vivo* and made two important observations (85–87). First, a striking diversity in number of progeny (over 1000-fold) from individual clones was observed, indicating a remarkable degree of variation in the naïve T cell responses. Whether this variation was the result of cell intrinsic programing of the naïve precursor, stochastic responses to activation, or differences in the microenvironment, was not clear. Second, even within individual clones, disparity in fate decisions was observed with some naïve precursors giving rise to uniform progeny, and others giving rise to progeny that had variable effector and memory characteristics. The data from one of these studies assessed 304 possible models for progression between naïve, effector, central memory and effector-memory states, and found only two of the models to fit their data, one of which allowed for ACD in the control of cell fate and the other did not (86). To support the notion that decision making could occur at multiple stages of T cell activation, limiting dilution, and short term progeny analysis demonstrated that T cell fate determination occurs before, during, and after the first T cell division (30).

### Is there evidence of polarity in dividing T cells?

It is now well established that in mature T cells, activation of the TCR triggers recruitment of polarity proteins (Scribble, Dlg1-4, PKCζ) to the immunological synapse (36, 88–93). Chang and colleagues contributed the first of steadily mounting evidence that mature T cells polarized polarity proteins during mitosis (94). Mitotic CD8^+^ T cells undergoing their first division following *Listeria* infection demonstrated asymmetric polarization of several polarity proteins including Scribble and PKCζ, the cell fate determinant Numb, and surface molecules important for T cell function such as CD8. This asymmetry was dependent upon the adhesion molecule, ICAM-1, and when populations of daughter T cells from the first division were sorted on the basis of differential CD8 expression and injected into *Listeria*-infected mice, mice receiving daughter cells with lower surface CD8 cleared the delayed infection more efficiently (94). This suggested that ACD could control memory differentiation in CD8^+^ T cells, although it has not yet been determined whether the disparate CD8 levels were a direct consequence of ACD. The finding that the transcription factor, T-bet, was asymmetrically partitioned into the daughters preferentially expressing CD8 provided support for the notion that ACD controls key fate determinants for effector memory decisions (95). This study also demonstrated that CD4^+^ cells display polarity at mitosis (95), and further work by the Reiner group showed that CD8 memory T cells can reinitiate ACD after rechallenge (96). Work by Palmer and colleagues also showed ACD of CD8^+^ T cells, and further demonstrated that peptide affinity can determine the extent of asymmetry during effector differentiation, and that the extent of asymmetry correlated with pathology (97).

These *in vivo* studies together provided the first indications that T cells can undergo ACD. The necessity for fixed staining of cells extracted from lymph nodes, however, means that the context of the cell division is not apparent. Without a defined cue, it is not possible to discriminate between ACD and asymmetry due to stochastic distribution of proteins at the time of division. For instance, it has not been possible to observe in these *ex vivo* experiments whether the dividing cell was attached to an antigen presenting cell to directly observe the subsequent behaviors of each T cell daughter. To address some of these issues, we have established an *in vitro* assay in which divisions can be observed in the context of interactions with the antigen presenting cell (98). In this system, T cell ACD required sustained contact with the antigen presenting cell but not a sustained immunological synapse. ACD of naïve T cells utilized conserved mechanisms, involving the Par3, Scribble, and Pins complexes to orchestrate spindle orientation. The cell fate determinant Numb was also localized asymmetrically, and disruption of mitotic spindle orientation caused mislocalization of Numb as well as altered memory and effector T cell fate ratios. Interestingly, there were several differences in protein asymmetry in this study and the *ex vivo* analyses described above. These include that the TCR and associated proteins were no longer polarized at the time of division in the *in vitro* system, and differences in the pole to which Numb was recruited. These differences might reflect differences in the experiments, such as *ex vivo* versus *in vitro* analysis, and the use of different transgenic systems and/or the use of Cytochalasin in the *ex vivo* experiments.

It would not be at all surprising for T cell ACD to be highly context dependent, with both qualitative and quantitative differences in ACD depending upon the context of T cell activation. This notion is supported by the study by Palmer and colleagues, in which different peptide ligands caused different degrees of asymmetry in the dividing cells (97). To further complicate the picture, *in vivo* imaging has suggested that interactions with the dendritic cell are transient around the time of division (99, 100), and that homotypic adhesions at this time can play a key role in fate determination (101, 102). Perhaps, therefore, some or all of the *ex vivo* dividing cells that exhibited asymmetry (94, 97) were polarized as a result of homotypic adhesions, which also depend upon ICAM-1 (101). A scenario in which ACD of CD8^+^ T cells could be qualitatively or quantitatively altered depending upon interactions with antigen presenting cells or other lymphocytes is compatible with the requirement that naïve CD8^+^ T cells must integrate many signals to orchestrate a robust but fine-tuned response to antigen presentation (103).

### What is the phenotype of polarity-deficient T cells?

Initial studies utilizing knockdown approaches to reduce the expression of Scribble complex proteins suggested that they played important roles in the development and function of T cells. T cells with reduced Dlg1 and Scribble showed impaired polarity and signaling in response to antigen presentation (78, 89, 90, 104), and impaired regulatory T cell function (105) as well as the developmental defect described above (77). In contrast, the analysis of T cells from mice with deleted polarity genes has shown either no, or very subtle, phenotypes. Expression of kinase dead forms of aPKC results in a reduction of polarization during migration and scanning (106), yet mice deficient in the atypical PKC isoforms PKCζ or PKCι/λ have an intact mature T cell repertoire, and normal responses with the exception of a defect in Th1 responses (56, 107–110). Mice deficient in Par1b exhibit alterations in CD44 expression on CD4 T cells, which might reflect aberrations in memory development, and this correlates with an involvement of Par1b in T cell polarity (111, 112). Three independently generated mice deficient in Dlg1 also exhibited normal T cell development and function, although again a defect in Th1 responses was observed in one mouse (68). Interestingly, the Th1 defect was observed in acute knockout (gene deletion driven by the CD4 promoter) and knockdown T cells, but not in T cells where the gene had been deleted in HSC, suggesting that compensatory mechanisms can occur during development to mask polarity-deficient phenotypes (68). In another study, Dlg1-deficient mice showed normal development and proliferative response, but a subtle change in the expression of CD44, 10 days after immunization, suggestive of a skewing of central and effector memory responses that was supported by differences in IL-2 production in immunized mice (113). Similarly, T cell development in Scribble and Lgl1 deficient mice was normal, as were the responses of Scribble-deficient mice to an influenza infection (63). Together, these studies indicate that the polarity proteins are not essential for HSC, T or B cell development and function, but that subtle effects can arise under some circumstances (Table 1).

A model for T cell ACD fate that links these findings could be as follows (Figure 3D). The polarity cue could derive from an interaction between the T cell and the antigen presenting cell or from homotypic interactions. A sustained immunological synapse may not be needed, but other molecules on the antigen presenting cell or the homotypic T cell, or chemokines might provide polarity cues. Quantitative and qualitative aspects of polarity at mitosis could be influenced by several factors such as the affinity of the TCR-MHC interaction, the duration of contact with the antigen presenting cell, the availability of other T cells for homotypic interactions. Partitioning of molecules, such as Numb and T-bet, differentially into one daughter cell would then cooperate with other signals from the microenvironment to fine tune the differentiation response.

## Future/Concluding Remarks

**Figure 4:**
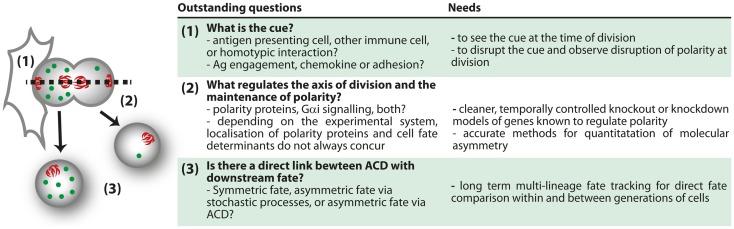
**Outstanding questions for ACD**.

Much more work is needed to reconcile the differences in phenotypes between different studies of polarity-deficient mice, and to determine whether or not immune defects in polarity-deficient mice are due to defects in ACD. The effect of knockout or knockdown of several polarity regulators has now been assessed, and the general picture is that the most striking phenotypes occurred with acute knockout or early in development, with emerging evidence that compensatory mechanisms can occur with time. Furthermore, no publications yet have indicated a correlation between these defects and evidence of asymmetry at mitosis, so it is not possible to definitively ascribe any of the phenotypes to a defect in ACD. In support of a role for ACD in immune cell development and function, some correlations are now emerging in which alterations in ACD are associated with alterations in fate. In this light, the relationship between ACD and pathology discovered by the Palmer group (97) is very encouraging. Similarly, inhibition of aPKC by the drug aurothiomalate (“Gold”) altered both ACD (polarization of Numb in dividing cells) and effector:memory ratios in our *in vitro* study (98). The loss of ICAM-1 also correlates with disruption in ACD and alterations in T cell fate (94), although the multifaceted role of ICAM-1 in effector and memory differentiation (114) complicates interpretation of this observation. Even the phenotypes from direct deletion of a polarity protein must be interpreted with caution, as these effects might be attributed to either ACD or the role of polarity proteins in the formation and function of the immunological synapse and downstream signaling (70, 76, 89, 90, 105). More acute knockouts, identification of genes that influence polarity at mitosis but not earlier (perhaps by regulating spindle orientation), and more extensive correlations of asymmetry and fate are required to fill these gaps in our knowledge (Figure 4).

Ultimately, definitive evidence of the impact of ACD on fate determination in lymphocytes will require more extensive integration of the three aspects of ACD, such that bifurcation of fate and polarity at division can be directly correlated. Several factors need to be taken into account in this endeavor. First, evidence to date suggests that ACD of lymphocytes is not uniformly adopted, but seems to arise in a fraction of the cells measured. Disruption of ACD would most likely impact upon some, but not all, the progeny of the population. Second, given the complexity of lymphocyte fate determination, and the many external cues that can influence lymphocyte fate determination, it seems that ACD of lymphocytes would more likely modify than determine fate decisions. Third, fate choices in lymphocytes often emerge incrementally over several generations, so measurements of the fate decisions made by each daughter cell must also be performed over a protracted period of time.

Such studies are now conceivable using *in vitro* approaches. Time lapse imaging of the process of division has been performed (95), and this type of approach can now take advantage of the rapid development in methods of quantification and duration of imaging (115–119). With these tools, it will be possible to directly observe how asymmetry at division can impact upon lymphocyte fate determination. Although it is unlikely that *in vivo* imaging will enable long-term fate tracking in the near future, the ability to observe cells over several hours (99, 100), and to track protein distribution (120) *in vivo*, will yield important information regarding the physiological context in which ACD can be observed. These approaches, combined with others such as the long-term *in vitro* time lapse imaging and the *ex vivo* analysis pioneered by the Reiner group (94), will together enable a comprehensive understanding of the mechanisms and roles of ACD in lymphocytes. The bulk of the research so far has been performed in CD8^+^ T cells, but many other aspects of lymphocyte differentiation and function might also involve ACD. Tracing the progeny of a single cell *in vivo* using approaches such as cellular barcoding, which have already provided evidence of fate bifurcation in the response of CD8^+^ T cells (85–87) are likely to yield important new information regarding the most physiologically relevant situations in which to look for ACD. Interestingly, a recent barcoding study argued against a clear bifurcation of fate in HSC (49). With the creation of more suitable knockout models, such as acute disruption of spindle orientation, new phenotypes might further highlight the systems in which ACD is most likely to play a physiological role (Figure 4). By combining all these approaches, a clearer picture of the mechanisms and consequences of ACD in lymphocytes is probably not too far away.

## Author Contributions

Kim Pham, Faruk Sacirbegovic, and Sarah M. Russell all wrote the manuscript.

## Conflict of Interest Statement

The authors declare that the research was conducted in the absence of any commercial or financial relationships that could be construed as a potential conflict of interest.
